# Evolutionary Analysis of Cystatins of Early-Emerging Metazoans Reveals a Novel Subtype in Parasitic Cnidarians

**DOI:** 10.3390/biology10020110

**Published:** 2021-02-03

**Authors:** Pavla Bartošová-Sojková, Jiří Kyslík, Gema Alama-Bermejo, Ashlie Hartigan, Stephen D. Atkinson, Jerri L. Bartholomew, Amparo Picard-Sánchez, Oswaldo Palenzuela, Marc Nicolas Faber, Jason W. Holland, Astrid S. Holzer

**Affiliations:** 1 Biology Centre, Institute of Parasitology, Czech Academy of Sciences, 37005 České Budějovice, Czech Republic; jiri.kyslik@paru.cas.cz (J.K.); gema.alama@gmail.com (G.A.-B.); amparo.picard@csic.es (A.P.-S.); astrid.holzer@paru.cas.cz (A.S.H.); 2 Faculty of Science, University of South Bohemia, 37005 České Budějovice, Czech Republic; 3 Department of Life Sciences, Natural History Museum, London SW7 5BD, UK; ash.hartigan@gmail.com; 4 Department of Microbiology, Oregon State University, Corvallis, OR 97331, USA; stephen.atkinson@oregonstate.edu (S.D.A.); jerri.bartholomew@oregonstate.edu (J.L.B.); 5 Fish Pathology Group, Instituto de Acuicultura Torre de la Sal (IATS-CSIC), 12595 Castellón, Spain; oswaldo.palenzuela@csic.es; 6 Scottish Fish Immunology Research Centre, Institute of Biological and Environmental Sciences, University of Aberdeen, Aberdeen AB24 3UU, UK; marc.faber@moredun.ac.uk (M.N.F.); j.holland@abdn.ac.uk (J.W.H.)

**Keywords:** cysteine protease inhibitor, stefin, signal peptide, parasite, phylogenetic analysis, diversification, protein structure

## Abstract

**Simple Summary:**

Cysteine protease inhibitors (cystatins) are molecules that play key protective roles in protein degradation and are involved in the immunomodulation of host responses to parasites. Little is known about the cystatin gene repertoire, evolution, and lineage-specific adaptations of early-emerging metazoans. Using bioinformatics searches, we identified orthologues of cystatins in basal animal lineages including free-living and parasite taxa. We aimed to explore whether their cystatin gene repertoire and evolution follow similar patterns recognized for derived metazoans and whether the modifications are linked to the organism’s life history. We revealed that cysteine protease inhibitors from early-emerging animal groups are highly diverse, with modifications in gene organization and protein architecture. A new subtype of cystatins was discovered in the parasitic cnidarians, the Myxozoa, which has so far been only reported for a group of derived animals: trematode flukes. We set out hypotheses to describe the driving forces for the origins of this unique cystatin subtype and propose evolutionary scenarios elucidating the current existence of cystatins in the Metazoa, especially in their early-emerging lineages. Our research identified molecules for which future functional studies may help to identify their roles in host–parasite interactions and for the parasite itself.

**Abstract:**

The evolutionary aspects of cystatins are greatly underexplored in early-emerging metazoans. Thus, we surveyed the gene organization, protein architecture, and phylogeny of cystatin homologues mined from 110 genomes and the transcriptomes of 58 basal metazoan species, encompassing free-living and parasite taxa of Porifera, Placozoa, Cnidaria (including Myxozoa), and Ctenophora. We found that the cystatin gene repertoire significantly differs among phyla, with stefins present in most of the investigated lineages but with type 2 cystatins missing in several basal metazoan groups. Similar to liver and intestinal flukes, myxozoan parasites possess atypical stefins with chimeric structure that combine motifs of classical stefins and type 2 cystatins. Other early metazoan taxa regardless of lifestyle have only the classical representation of cystatins and lack multi-domain ones. Our comprehensive phylogenetic analyses revealed that stefins and type 2 cystatins clustered into taxonomically defined clades with multiple independent paralogous groups, which probably arose due to gene duplications. The stefin clade split between the subclades of classical stefins and the atypical stefins of myxozoans and flukes. Atypical stefins represent key evolutionary innovations of the two parasite groups for which their origin might have been linked with ancestral gene chimerization, obligate parasitism, life cycle complexity, genome reduction, and host immunity.

## 1. Introduction

Members of the cystatin superfamily (I25), also known as cystatins, are major regulators of the activity of papain-like cysteine proteases and legumain-type proteases. These endogenous protease inhibitors are widely distributed in all domains of life. Based on sequence homology, the cystatin superfamily comprises three main sub-families: types 1 –3. Type 1 cystatins (stefins, I25A) are low-molecular-weight intracellular single-domain proteins generally lacking disulfide bonds and signal peptides. Within the stefins, an additional subtype, unusually featuring a signal peptide, is recognized exclusively in liver and intestinal flukes (Trematoda: Digenea) [1,2,3,4]. Type 2 cystatins (I25B) are mainly extracellular single-domain proteins containing two disulfide bonds and a signal peptide, while type 3 cystatins (kininogens, I25C) are large multi-domain proteins [5,6,7]. 

Cystatins are involved in the regulation of key physiological processes, such as the protection of cells from proteolysis [5,6,7]. In parasites, cystatins are involved in immunomodulation of host cells and represent important immunogenicity and pathogenicity factors involved in host–parasite interactions [8,9,10,11,12,13]. They facilitate parasite survival within the host [14,15] by inhibiting host proteases and by interfering with antigen processing and presentation [16,17,18,19]. The increased capacity of parasite cystatins to induce anti-inflammatory cytokine IL-10 [16,17,20] was suggested to be an adaptation to the parasitic lifestyle [21,22]. These molecules have been used as effective markers in diagnosis and vaccine development and as chemotherapeutic targets against various parasitic infections [23,24,25,26,27]. Recently, cystatins have also been suggested as candidates for immunotherapies for acute and chronic inflammatory diseases [28] and cancer [29].

The diversity and evolution of the cystatin superfamily is well-documented in prokaryotes and certain eukaryotic lineages [30,31,32,33]. Within the Metazoa, phylogenetic affinities and representation of cystatins have been investigated predominantly in the derived lineages (Bilateria) including both free-living [34] and parasite taxa, e.g., various helminth [2,10,35,36,37,38] and arthropod groups [39,40,41,42]. According to the current knowledge of cystatin evolution, the stefin and type 2 cystatin lineages were already present in the eukaryotic ancestor. Stefins have remained structurally similar to the ancestral form and are present across most metazoan groups. In contrast, type 2 cystatins greatly diversified, primarily in derived metazoans (Vertebrata), and have been lost entirely in some early (Placozoa) and derived (Acoela, Tardigrada, Hemichordata, etc.) metazoan groups. Multi-domain cystatins have been reported exclusively from several bilaterian groups [30]. Nevertheless, the picture of cystatin evolution in the Metazoa is incomplete, as early-emerging animal lineages (Porifera, Ctenophora, Placozoa, and Cnidaria) are greatly underexplored in this respect and completely underrepresented with regard to parasitic lineages. To date, only a few early-emerging animal taxa were included in the studies of cystatin evolution [30,34], from which none were parasites. Thus, further data are needed from these ancient animal lineages, including those with parasitic lifestyles, to improve our evolutionary understanding and to explore potential linkage to the organism’s life history.

Basal metazoans mostly comprise free-living species, but at least eight independent parasite lineages of early-emerging Metazoa are known, with all but one in Cnidaria [43]. Recently, next-generation sequencing (NGS) data have become available for three of these parasite lineages, i.e., two *Edwardsiella* spp. [44,45], *Polypodium hydriforme* [46,47], and several species of the large group Myxozoa [47,48,49,50,51,52,53,54]. While *Edwardsiella* and *Polypodium* parasitize their hosts exclusively during the larval stage of their development [45,55], the microscopic myxozoans are an entirely parasitic group with complex life cycles [56]. Members of the specious class Myxosporea are primarily known from fish (intermediate hosts) and annelids (definitive hosts). The more primitive Malacosporea use fish and freshwater bryozoans as hosts [57]. Myxozoans are an evolutionary old and fast-evolving group, evidenced by their highly derived morphology and seen in genome phylogenies [46,47,51,53,58]. Their genomes are the smallest amongst all animals due to evolutionary transition to parasitism and the associated extreme reduction of body complexity from free-living cnidarians [47].

To explore the gene repertoire, gene organization, and structural features of cystatins in phyla at the base of the metazoan tree, we mined 110 genomes and transcriptomes of 58 early-emerging animal species. We conducted phylogenetic analyses using newly obtained and published cystatin sequences of basal and derived metazoans to provide insights into the origin, evolution, and structural diversification of this group of inhibitors at the time when this superfamily emerged and expanded. We investigated the amino acid sequence structure and phylogenies of cystatins from free-living and parasite taxa to identify structural modifications, which potentially represent lineage-specific adaptations related to parasitic lifestyles. Our investigation thus aimed to reveal whether gene repertoire and evolution of cystatins in early-emerging animals follow similar patterns recognized for derived metazoans and whether the modifications are linked to the organism’s life history.

## 2. Materials and Methods

### 2.1. Data Mining

Representatives of basal metazoan lineages (Placozoa, Porifera, Ctenophora, and Cnidaria) were selected for mining of cystatin homologues from the NGS data available from GenBank, other public databases, or produced in our laboratories (110 datasets of 58 species; Table S1). The samples included free-living and parasite taxa, particularly Cnidaria, i.e., *Polypodium hydriforme*, *Edwardsiella lineata, E. carnea*, and all available myxozoans (13 species). We used published the cystatin homologues of metazoans from basal animal species [30,53] for the cystatin homology search. If no homologues were found in a species in the first blast, a repeated search was applied by utilizing the sequences of homologues found in related species in the first iteration as additional queries for the next search.

We used a combined search strategy using both motif- and sequence similarity-based methods to identify target molecules. The search was performed using the tBLASTn algorithm with the E-value cutoff set to 10^−5^. This relatively low threshold was selected based on low sequence similarity among our queries and subjects (approximately 30–40%). The mined sequences were examined for the presence of typical conserved motifs of the cystatin domain (G; Q-x-V-x-G; LP/PW; YF [30,59]), by searches against different databases (GenBank, MEROPS peptidase database [60]) including the reciprocal blast analysis, and by their positioning in our preliminary phylogenetic analyses. Detailed homology searches were performed using the hmmrscan algorithm of HmmerWeb version 2.32.0 software [61] against protein family databases: Pfam, CATH-Gene3D, PIRSF, Superfamily, TIGRFAM, and TreeFam. For parasites, the potential contaminating sequences of host or other origin were identified and filtered out by blast matches with reference NGS data and by phylogenetic clustering with contaminant species homologues.

### 2.2. Verification of Selected Cystatin Sequences 

Full gene sequences of the cystatin superfamily homologues mined from unpublished genomes and transcriptomes (Table S1) were verified by PCR and by sequencing from DNA and/or cDNA templates using species-specific primers designed in this study, if possible, in the 5’and 3’UTR regions (details in Methods S1 and Table S2). PCR was used to obtain the stefin gene sequence of *Buddenbrockia plumatellae* (Methods S1 and Table S2) as mining of the published *Buddenbrockia* EST database (Table S1) did not return any cystatin hits. PCR-verified sequences were deposited in GenBank under the accession Nos. MT127416–MT127426 and MW498387–MW498390 (Table S2). The positions of the introns were assessed by comparison of the sequences of DNA vs. cDNA origin and, if possible, by comparing sequences mined from the genomic vs. transcriptomic data.

### 2.3. Phylogenetic Analyses

We compiled and analyzed a sequence dataset (128 taxa) of both newly identified and published cystatins. The dataset comprised Choanoflagellates (11 taxa), early-emerging animal lineages (58 taxa), more derived metazoans (58 taxa), and *Giardia* cystatin (outgroup) resembling the most ancestral eukaryotic cystatin [30] (Table S3). Ingroup taxa were represented by free-living species (72 taxa) and parasite species from basal metazoans (*Polypodium hydriforme*, *Edwardsiella* spp., and myxozoans; 16 taxa) and bilaterians (trematodes, cestodes, nematodes, monogeneans, ticks, and mites; 39 taxa) (Table S3). Multi-domain cystatins were omitted from the phylogenetic analyses due to difficulties with their alignment. 

We aligned 329 amino acid cystatin sequences in MAFFT v7.017 implemented in Geneious v8.1.3 [62] using the iterative refinement by the G-INS-i strategy that resulted in an alignment of stefins and type 2 cystatins according to topological equivalence in tertiary structure (see Dataset S1 of [59]). Nonhomologous regions at the beginning (e.g., signal peptides) and end of the alignment were trimmed so the final alignment comprised 213 positions, principally corresponding to the conserved cystatin domain (Pfam: PF00031) (Dataset S2). The phylogenetic trees were reconstructed using the maximum likelihood (ML) and Bayesian inference (BI) methods. ML analysis was performed in RAxML v7.0.3 [63], using the WAG + G model selected by ModelFinder implemented in IQ-TREE webserver [64]. Indels were treated as unknown characters. Bootstraps were based on 1000 replicates. BI analysis was performed in MrBayes v3.2.7av1 [65] using the WAG model of evolution, with 24 million generations sampled at intervals of 1000 trees until the standard deviation from split frequencies was below 0.05. The burn-in period represented the default initial 25% of all generations. The trees were visualized in Geneious v8.1.3 and graphically modified in Adobe Illustrator CS5.

### 2.4. Comparison of the Protein Architecture

Cystatin diversity on the amino acid sequence level was analyzed in terms of critical motifs, amino acid conservation, presence/absence of a signal peptide and disulfide bridges, and modifications that might potentially affect the cystatin inhibitory activity. Disulfide bonds were predicted using the DISULFIND webserver [66]. Signal peptides were predicted using the SignalP v5.0 webserver [67]. Animal group-/cystatin type-specific sequence motifs were identified by comparison of primary structures of cystatin amino acid sequences in the WebLogo v3 software [68] using extractions of the alignment used for the phylogenetic analyses. 

## 3. Results

In this study, we retrieved 184 homologues of cystatins (104 stefins and 80 type 2 cystatins) from the NGS data of early-emerging metazoans (Table S1). Of these, we verified with PCR the identity of 15 homologues mined from unpublished datasets (Table S2).

### 3.1. Cystatin Gene Repertoire and Diversity in Early-Emerging Metazoans

**Figure 1:**
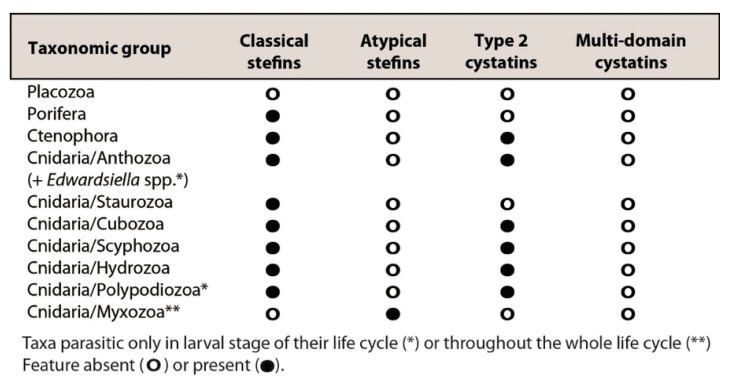
Repertoire of cystatin superfamily genes in early-emerging metazoan lineages.

**Figure 2:**
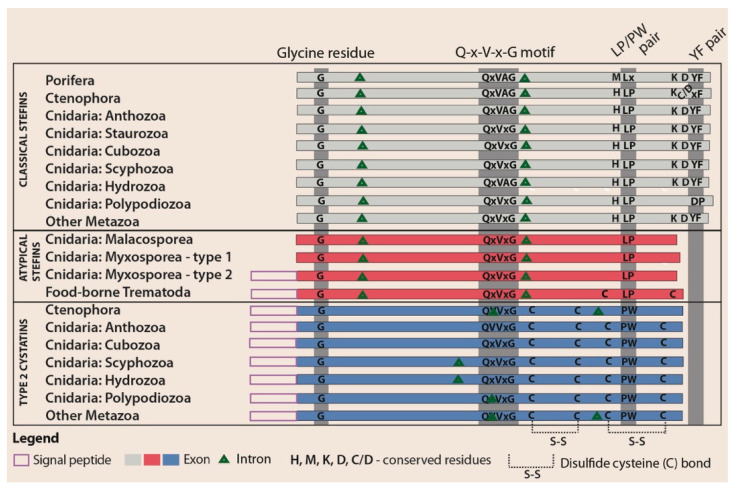
Schematic comparison of the gene architecture and amino acid structure of metazoan cystatins: for myxozoans, information is shown separately for Malacosporea and Myxosporea.

We observed a large variation in the representation of cystatin subclasses in the investigated animal groups (Figure 1). Type 2 cystatins were missing in several groups (Placozoa, Porifera, Cnidaria: Staurozoa, and Myxozoa). Stefins were present in all but one (Placozoa) basal metazoan lineage. We found a separate group of stefins in myxozoan parasites, designated here as “atypical stefins”, which differed in sequence structure to classical metazoan stefins (see Section 3.3 and Figure 2). No multi-domain cystatins could be identified in basal metazoans (Figure 1).

We found that basal metazoans had a diverse cystatin gene repertoire: most investigated species had multiple cystatin homologues with significant sequence differences, even on the intraspecies level (i.e., several divergent sequences of type 2 cystatin and/or stefin homologues in the genome of a single species; Table S1). Exceptionally, certain species (e.g., cnidarians *Ceratonova shasta* and *Physalia physalis*) only possessed single type 2 cystatin and/or stefin homologue.

All mined cystatin homologues are listed either as new GenBank entries (for PCR-verified sequences mined from data unpublished at the time of the homology searches) or as Genbank contig/scaffold identifiers for sequences mined from published data (Table S2 and Dataset S1).

### 3.2. Phylogeny of Metazoan Cystatins did not Mirror Animal Phylogeny and Was Highly Diverse within Individual Groups

**Figure 3:**
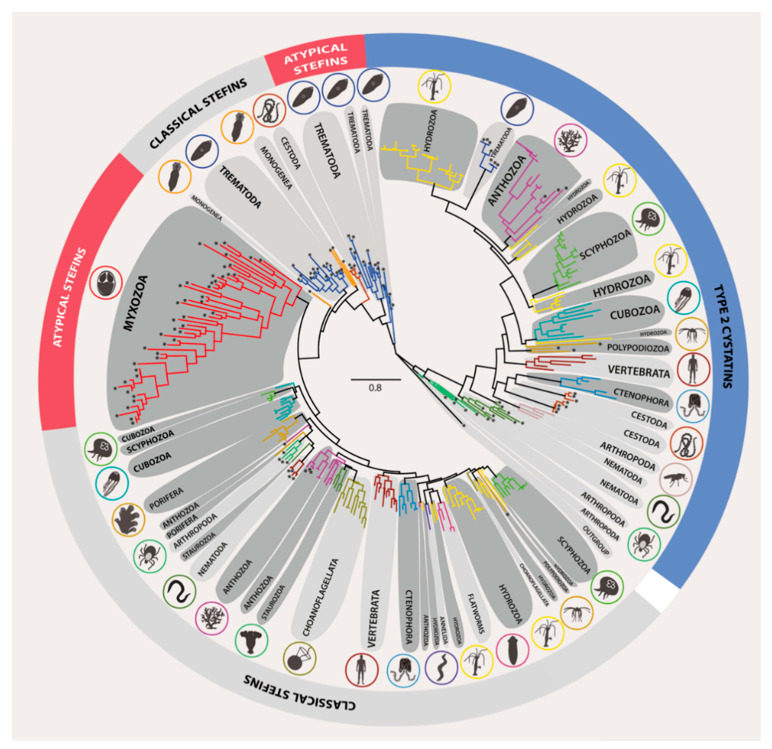
Maximum likelihood phylogenetic tree of the cystatin superfamily represented in the Metazoa and Choanoflagellata. *Giardia* cystatin was used as the outgroup. The basal metazoan groups are depicted in dark grey shaded bubbles, while other groups are shaded light grey. Cystatin homologues from parasite taxa are labelled by asterisks at the tip of the nodes. The detailed ML and BI phylogenetic trees featuring taxa names and all nodal supports are provided in Figure S1.

The clustering of cystatins in both ML and BI analyses did not follow the generally accepted trends of organismal phylogeny, as early-emerging animal lineages did not occupy basal positions in our trees (Figure 3 and Figure S1) as commonly known from other phylogenetically informative genes., e.g., 18S rDNA. Based on differences in the primary amino acid sequence structure, cystatins clustered into a single weakly supported stefin clade (ML/BI = 48/0.75) while the remainder of sequences clustered within the type 2 cystatin clade (ML/BI = 59/0.92). The stefin clade included a mixture of classical and atypical stefin subclades with weakly supported interrelationships. Classical stefin subclades comprised all Metazoa except the myxozoans, while atypical stefin subclades were formed exclusively of certain early-emerging (myxozoans) and more derived (trematodes) parasite animal lineages. Unlike other metazoan stefins, those of myxozoans were extremely divergent in their sequences, thus creating long branches (Figure 3 and Figure S1).

Both stefins and type 2 cystatins clustered into subclades that paralleled classic taxonomy. However, interclade relationships of these taxonomy-defined subclades were weakly supported/not resolved due to polytomies (Figure 3 and Figure S1) and did not always reflect the organismal phylogeny. Indeed, some representatives formed monophyletic taxonomic groups, e.g., Ctenophora, Porifera, Cubozoa, and Myxozoa, while some cnidarian groups (Polypodiozoa, Staurozoa, Scyphozoa, Anthozoa, and Hydrozoa) split into multiple subclades not related by classic taxonomy. We attributed this splitting to (i) sequence differences among species of the same taxonomic group (e.g., hydrozoans *Hydra*, *Podocoryna*, *Physalia*, *Velella*, and *Porpita* spp. clustering into independent type 2 cystatin clades) or (ii) the occurrence of paralogues (e.g., stefin out-paralogues of the scyphozoan *Aurelia aurita* present in two distinct groups). Some of the out-paralogous clades additionally contained multiple sequences from single species (e.g., stefin in-paralogues of *A. aurita* ranging up to 37.7% amino acid sequence divergence) (Figure 3 and Figure S1). 

### 3.3. Structural Modifications of Cystatin Homologues in Basal Metazoans

The results of structural analyses aimed at identifying intron number and positions, conserved regions, and the presence/absence of signal peptides and cysteine bonds were combined to create the comparative scheme of cystatin structural motifs (Figure 2).

We identified that introns in cystatin nucleotide sequences of early-emerging animal lineages varied in number (0–2) and location in type 2 cystatins while stefins contained two introns with conserved positions (Figure 2). All introns were delimited by typical GT/AG sequence boundaries at the exon–intron junction.

In amino acid sequence data from our alignment of basal animals (Dataset S2), we observed four conserved regions important for interaction with proteases, as found previously with derived metazoans [30,59]: (i) a glycine residue within the amino-terminal “trunk”, (ii) a Q-x-V-x-G motif within the first hairpin loop, (iii) the PW (type 2 cystatins) or LP (stefins) motifs within the second hairpin loop, and (iv) a tyrosine residue located in the relatively conserved D-x-L-x-Y-F carboxy terminus of stefins. We observed two novel conserved residues in stefins of both early-emerging and derived metazoans: (i) a histidine residue located seven amino acids before the LP pair towards the N-terminus and (ii) a lysine residue situated eight amino acids before the C-terminal conserved tyrosine residue (Figure 2 and Dataset S2). 

While the amino acid structure of the first two key activity regions (G, Q-x-V-x-G) was conserved among all sequences, several modifications were seen in other regions. All sponge (Porifera) stefins showed a characteristic amino acid replacement of the LP pair to LD (or LS in one case). Substitution of the conserved C-terminal tyrosine residue by non-aromatic valine was observed in two homologues. The conserved histidine residue located near the LP pair was commonly replaced by methionine or, exceptionally, by histidine or tryptophan (both observed once) (Dataset S2).

Stefins and type 2 cystatins of comb jellies (Ctenophora) had the typical sequence motifs defined for these molecules in other metazoans. In a few taxa, we observed substitutions of the conserved cystatin PW pair by AW and stefin C-terminal tyrosine residue by proline or histidine (Dataset S2).

Cnidarian cysteine protease inhibitors showed a wide variety of modifications (Figure 2 and Dataset S2). Cubozoans were the only group for which conserved stefin regions had the classical organization, and their type 2 cystatins differed in a few cases by replacement of the PW motif with SW or KF. Stefins of hydrozoans and scyphozoans showed single substitutions of LP to FA or LK and single alterations of C-terminal tyrosine to aromatic phenylalanine, and many of the type 2 cystatins had substitutions of PW with AW, RW, or SW and exceptionally with PF or PL. Staurozoan stefins had C-terminus modifications, where the tyrosine residue was substituted with histidine or was missing. In anthozoans, type 2 cystatins had alterations of PW to SW or GW, while a few stefins had substitutions of LP with FD or FS and replacement of the C-terminal tyrosine residue to proline or histidine. 

The most notable modification we found was in two parasitic cnidarian groups Polypodiozoa and Myxozoa. While the majority of type 2 cystatins of *P. hydriforme* had the typical conserved motifs, its stefin was highly modified, with the LP pair substituted with LS and the C-terminal conserved D-x-L-x-Y-F motif replaced with a completely different pattern lacking a tyrosine residue. While this stefin retained the histidine found near the LP pair, the conserved lysine located near the C-terminal was replaced by leucine. Myxozoans, as representatives of early-emerging metazoans, had atypical stefins, which completely lacked the D-x-L-x-Y-F region at the C-terminus and had the normally conserved histidine and lysine residues replaced by other residues. Stefins of an evolutionary older myxozoan subgroup, the Malacosporea, retained the original LP pair, but some stefins of the specious and more derived myxozoan group, the Myxosporea, had replaced this motif with LS, LY, LR, FK, or SA (Figure 2). We could not find classical stefins or type 2 cystatins in any myxozoan (Figure 1).

In general, signal peptides in cystatins of basal metazoan lineages followed established patterns: they were present in type 2 cystatins and absent in classical stefins. The only exceptions were some myxosporean stefins that carried a signal peptide (Figure 2). Interestingly, the pattern of signal peptide absence (in type 1 atypical stefins) or presence (in type 2 atypical stefins) was observed even at the intraspecies level. For example, some myxosporeans coded exclusively for stefins without a signal peptide (i.e., *C. shasta* and *Sphaeromyxa zaharoni*) while others presented multiple stefin genes that all carried the signal peptide (i.e., *Myxobolus pendula* and *M. cerebralis*) or had genes that represented both variations (i.e., *Enteromyxum leei*, *E. scophthalmi*, *Kudoa iwatai*, *Thelohanellus kitauei, Myxidium lieberkuehni* and *Sphaerospora molnari*) (Figure 2 and Dataset S1).

As typical for type 2 cystatins, two conserved disulfide bridges were identified in all basal metazoans with the exception of homologues found in ctenophorans, which were predicted to contain only one disulfide bridge. Classical stefins lacked disulfide bridges, as is typical for this cystatin subfamily type, and this pattern was also observed in atypical stefins (Figure 2).

### 3.4. Structural and Evolutionary Comparison of Cystatins of Parasitic vs. Free-Living Groups of Basal Metazoans: The Special Case of Myxozoa

The Myxozoa, the only obligate parasite group of early-emerging metazoans, possessed exclusively atypical stefins and lacked type 2 cystatins. Myxozoan stefins had characteristic differences in sequence to those of the other basal Metazoa (see Section 3.3) and clustered into a single clade with long branches (see Section 3.2). The two cnidarian lineages studied that are parasitic only in their larval stage (*E. lineata*, *E. carnea*, and *P. hydriforme*) possessed both classical stefins and type 2 cystatins, and no atypical stefins were found in these animals. Their stefins formed a distinct lineage that afterwards clustered with other cnidarians (*P. hydriforme*) or grouped with their respective anthozoan members (*E. lineata* and *E. carnea*). Similarly, clustering of “partially parasitic” cnidarians into distinct monotypic lineages (*P. hydriforme*) or within anthozoan lineages (*E. lineata* and *E. carnea*) was observed for type 2 cystatins (Figure 3 and Figure S1). No structurally unique molecules were identified in the entirely free-living basal metazoans.

## 4. Discussion

### 4.1. A Diverse Repertoire of Cystatins in Early-Emerging Metazoans

In this study, we identified the cystatin gene repertoire of early-emerging animals by mining homologues from 110 genomes and transcriptomes of basal Metazoa. We demonstrate that cystatins are widely distributed and highly diversified in current representatives of these ancient lineages and that the repertoire of cystatin superfamily genes differs widely among phyla. Each taxonomic group evolved multiple stefin and/or type 2 cystatin homologues, forming individual taxonomy-defined clades in the phylogeny. These paralogues probably arose by gene duplication [30] rather than by whole genome multiplication, as the only basal metazoans for which genome duplication has been suggested are members of the cnidarian genus *Acropora* [69]. Multiple out-paralogues found in cnidarians likely originated before cnidarian radiation while, in-paralogues, probably emerged after the speciation event. Bursts of sequence diversification likely gave rise to gene orthologues within each taxonomy-defined clade. These evolutionary processes resulted in the origin of diverse molecules with varying inhibitory and immunomodulatory functions in metazoans. 

Some taxonomic groups had a wide diversity of cystatins, while others lacked some or all cystatin subfamilies. For example, genes coding for cystatins were not identified in the placozoan genomes: two different strains of *Trichoplax* sp. [70] and *Hoilungia hongkongensis* [71], which was in concordance with findings of Kordiš and Turk [30] from the related species *Trichoplax adhaerens* [70,72]. Evidently, cystatins are not essential for the survival of these simple organisms despite the presence of cysteine proteases in them [71,72]. We suggest that, in placozoans, the functions of cystatins have been replaced by other types of inhibitors, such as structurally unrelated equistatin or serpins [70,71,72]. These proteins have been recognized in other organisms as potent inhibitors of cysteine proteases [73] or cross-class inhibitors of them [74].

### 4.2. Atypical Stefins: A Unique Type of Cystatins in Myxozoans and Some Trematodes

Interestingly, we found a wide diversity of both classical and atypical stefins in basal metazoans and we suspect that these highly divergent homologues may have different or novel functions, as suggested for the highly divergent myxozoans serpins (serine protease inhibitors [75]). Stefins structurally similar to those of myxozoans have been identified only from more derived bilaterians: intestinal and liver flukes (Trematoda: Digenea) [1,2,3,4]. While myxozoans possess only atypical stefins, these trematodes have cystatins from several superfamily subclasses (classical and atypical stefins, multi-domain cystatins [1,2,3,4,22,76]). The atypical stefins presumably arose independently in the two groups, possibly as a result of pressures from a parasitic life history; however, other parasites do not possess these molecules. For example, parasitic metazoans (monogeneans, nematodes, cestodes, ticks, and mites) [9,36,37,39,42] including blood trematodes [35] have only classical stefins and type 2 cystatins. Similarly, cnidarians *Polypodium* and *Edwardsiella* spp. with parasitic larval and free-living adult stages also have only classical stefins and type 2 cystatins. Thus, atypical stefins represent key evolutionary innovations of myxozoans and intestinal and liver trematodes. The likely function of atypical stefins in myxozoans and trematodes is to control unwanted proteolysis by cysteine proteases expressed by different parasite life stages and by the host [1].

Structurally, atypical stefins contain the characteristic stefin LP pair, which is modified in certain myxozoan stefins. Importantly, all atypical stefins lack the conserved D-x-L-x-Y-F carboxy-terminal part that is generally found in classical stefins but is absent from type 2 cystatins (Figure 2). As the tyrosine residue of the D-x-L-x-Y-F region is important for stabilization of the stefin-protease complex [59,77], the absence of this motif along with the modifications in the conserved LP pair may have functional implications in atypical stefins, e.g., their capability to inhibit a broader range of proteases, including those of host origin. The function of this C-terminal modification warrants future experimental studies as demonstrated previously for stefin mutants [77,78]. 

Additionally, we identified that the atypical stefins of some myxozoans and all trematodes have a signal peptide typical for type 2 cystatins (Figure 2). In some taxa, this feature correlates with extracorporeal protein secretion [3,79,80,81], but this is not consistent across the Metazoa, as not all proteins that have a signal peptide are exported out of the organism [1] and a large proportion of excreted/secreted products do not have a signal peptide [1,4,37,79,81,82,83]. Secreted proteins, including cystatins, are crucial for parasite protection from degradation by host cysteine proteases and for modulation of host immunity [9,10,11,16,79]. We postulate that some myxozoan stefins are secretory proteins with immunomodulatory roles, as has been suggested for the atypical stefin of the myxosporean *Thelohanellus kitauei* [53,84]. These molecules are likely responsible for the increased expression of the anti-inflammatory IL-10 gene in fish hosts infected with different myxosporean species [85,86,87,88,89,90] as an increased production of IL-10 in hosts infected by other parasites is elicited by cystatin-induced immunomodulation [16,20]. Similar to other parasites [23,24,25,26,27], we suggest that these proteins should be further explored in myxozoans as vaccine targets due to the lack of efficient strategies against these parasites in fish destined for human consumption [91,92].

We propose two mechanisms for the origin of atypical stefins: (i) structural modification of ancestral classical stefins by the acquisition of a signal peptide in some homologues and loss of the C-terminal and its tyrosine residue, or (ii) combination of the features of classical stefins (LP pair) and type 2 cystatins (signal peptides, missing C-terminal part) in chimeric genes. Chimeric genes are important in the evolution of genetic novelty and can allow organisms to adapt to novel environments (or hosts) by acquisition of novel functions [93,94]. Both myxozoans and trematodes are obligate parasites with complex life cycles that involve vertebrate and invertebrate hosts [8,95]. The independent origins of their atypical stefins might have been associated with the transition of a presumably free-living ancestor to obligate parasitism and the associated challenges of surviving within one or more hosts. Extending a recent hypothesis that invertebrates were the initial hosts for myxozoans and fish were incorporated into life cycles later [96], we postulate that myxozoans reduced their type 2 cystatins in the invertebrate host in the course of genome reduction due to parasitism [47] since invertebrates have limited immune capacity [97]. Ancestral superfamily cystatin types were lost in myxozoans as their roles were replaced by atypical stefins, which in trematodes have been shown to inhibit both parasite and host proteases [2,3]. Then, under novel pressures in adopted fish hosts, myxozoans remodeled classical stefins into the atypical ones to interfere with specific immune responses of fish and to enable novel behaviors such as migration within host tissues.

### 4.3. Obstacles to Reconstruction of Cystatin Phylogenies

In our analyses, the reconstruction of evolutionary relationships in the cystatin superfamily had good support for the more recent clades but not for deeper evolutionary nodes, as reported previously for this group of genes [30,35]. Unstable topology, polytomies (in BI), and low nodal supports in our phylogeny were due to low informative content from short protein length and high sequence divergence in the chosen genes [30,35]. However, despite generally poor support, a clear separation of ingroup taxa into distinct lineages defined by taxonomy and protein architecture revealed remarkable trends in the evolution of cystatins in early-emerging metazoans, with structurally unique molecules of potentially novel functions. Different branches appear to have evolved at different rates [98], especially in fast-evolving myxozoans [46,47,51,53,58]. Fast rates of evolution of the myxozoan genes likely caused the extraordinary sequence divergence of myxozoan stefins, which may represent functional diversification of these molecules. Phylogenetic clustering of atypical stefins within the myxozoan clade seems to some extent to follow myxozoan speciation and differentiation into the well-known myxozoan lineages (oligochaete-infecting, polychaete-infecting, *Sphaerospora sensu stricto*, and malacosporeans [96]) Resolution of these established myxozoan clades is low due to the undersampling of taxa and is probably compounded by diversification including a wide functional range of atypical stefins, with or without signal peptides, and with each particular lineage displaying specific sequence motifs and potential host organ-specific protease expression.

### 4.4. Hypothetical Evolution of Metazoan Cystatins

From their comprehensive analysis of prokaryotic and eukaryotic cystatins, Kordiš and Turk [30] concluded that the ancestor of the cystatin superfamily was intracellular and lacked a signal peptide and disulfide bridges (similarly to extant stefins). Initial gene duplication produced two ancestral eukaryotic lineages, stefins and type 2 cystatins, with the latter evolving from the stefin-like ancestor by acquiring cysteine residues, disulfide bridges, and a signal peptide. Later gene and domain duplications combined with deletions and insertions of genetic material resulted in diverse single- and multi-domain proteins with or without disulfide bonds and glycosylations [30]. 

**Figure 4:**
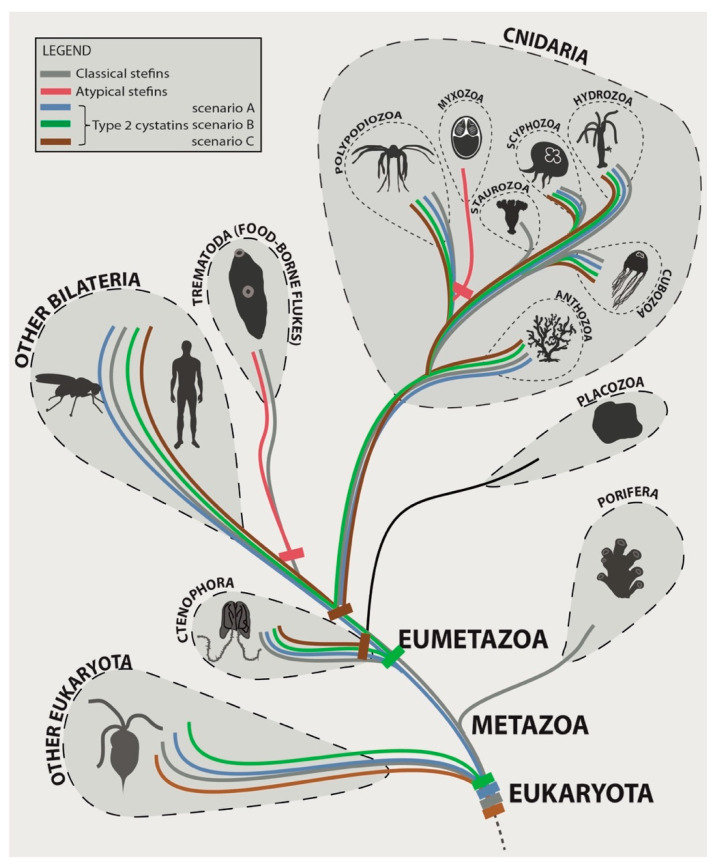
Hypothetical evolutionary scenarios of origins of stefins and type 2 cystatins in the Metazoa with a focus on early-emerging animal lineages: please note that the different colors for type 2 cystatins visualize the alternative scenarios of hypothetical evolution for the same cystatin subtype and not the sequence differences.

Based on our novel data and published records [30], we propose the following alternative scenarios for the evolution of single-domain cystatins in the Metazoa, with a focus on its basal lineages (Figure 4). After the split of the cystatin superfamily into the initial stefin and type 2 cystatin lineages in the ancestor of eukaryotes, stefins were retained in the common ancestor of the Metazoa and all its descendant lineages except Placozoa. Sequence diversification and lineage-specific adaptation of stefins occurred independently in parasites (Myxozoa and Trematoda), giving rise to atypical stefins. Type 2 cystatins were either (i) retained in the metazoan ancestor and its descendant lineages except for their independent loss in Porifera, Placozoa, some Cnidaria (Myxozoa, Staurozoa) and some bilaterians (liver and intestinal flukes) (scenario A in Figure 4); (ii) initially lost in the metazoan ancestor (or even before), reappeared in the ancestor of Eumetazoa, and were again lost in some descendant lineages (Placozoa, Myxozoa, Staurozoa, and some flukes) (scenario B in Figure 4); or (iii) initially lost in the metazoan ancestor (or even before) and reappeared independently in Ctenophora and in the ancestor of the lineage, leading to Cnidaria and Bilateria with some groups (Myxozoa, some flukes) having a secondary loss (scenario C in Figure 4). 

## 5. Conclusions

Stefins are considered relatively conserved genes with characteristics closer to the ancestral superfamily type, while type 2 cystatins are considered to have undergone a more complex and dynamic evolution through numerous gene and domain duplications [30]. In this study, we showed that early-emerging metazoan lineages were more prone to type 2 cystatin loss and that significant diversification of this type of molecules occurred only later in their evolution. Simultaneously, these ancient lineages retained stefin genes that were more dynamic with regard to structural modifications, with bursts of diversification creating atypical stefins in some obligate parasites with complex life cycles. Further research aimed at biochemical and functional characterization of the parasite cysteine protease inhibitors, especially of atypical stefins, is necessary to decipher the roles of these proteins in multi-host–parasite interactions. This knowledge will improve the understanding of the contributions of cystatins for parasite survival in the host causing disease and may reveal targets for therapeutant development against Myxozoa.

## Data Availability

The data presented in this study are openly available in Genbank (under the accession Nos. MT127416–MT127426 and MW498387–MW498390) and in the associated supplementary files.

## Supplementary Materials

The following are available online at https://www.mdpi.com/2079-7737/10/2/110/s1, Figure S1: Maximum likelihood and Bayesian trees with nodal supports; Table S1: Next-generation sequencing databases mined for cystatins; Table S2: List of myxozoan species with information on primers used for the PCR verification of stefin genes; Table S3: The list of groups/species used for phylogenetic analyses. Methods S1: Methodological details regarding the PCR verification of myxozoan stefins; Dataset S1: Fasta file of complete the amino acid sequences of cystatins present in the phylogenetic analyses; Dataset S2: Fasta-formatted alignment of the trimmed amino acid sequences of cystatins used for the phylogenetic analyses.
