# Supplementary material for: Evolutionary Analysis of Cystatins of Early-Emerging Metazoans Reveals a Novel Subtype in Parasitic Cnidarians
Source: Biology (Basel). 2021 Feb 3;10(2):110. doi: 10.3390/biology10020110 (PMC7913475; doi:10.3390/biology10020110)
Supplement: Supplementary file 1 [file biology-10-00110-s001.zip › supplements/Table S2. PCR verification primers.pdf]

Table S2. List of myxozoan species with information on primers used for PCR verification of stefin genes.

Myxozoan species	Host species	Host organ	Locality	Primer name and sequence	Amplified region (bp) from DNA/cDNA	GenBank Acc. nr.
Sphaerospora molnari	*Cyprinus carpio*	Blood, gills	Malá Outrata, Czech Republic	Sm1_5UTR_F: 5′-CGGACCGAGAGATTACGGTTA-3′<br>Sm1_5UTR_R: 5′-TTTTTAGGGACAAAAGTGAAAAGA-3′	395/359	MT127416
				Sm2_5UTR_F: 5′-GGATGGAAGATCAACGACCT-3′<br>Sm2_5UTR_R: 5′-GTTTTATGTGAATTTAGAACGAAAAGC-3′	524/450	MT127417
Ceratonova shasta (IIR genotype)	*Oncorhynchus mykiss*	Intestine, ascites	Willamette River, Oregon, USA	Cs_5UTR_F: 5′-CGCATGCCAATATATTATTATCCTA-3′<br>Cs_3UTR_R: 5′-TAGAAAGGAAATGTCATAGAAAACTCT-3′	356/313	MT127418
Myxidium lieberkuehni	*Esox lucius*	Urinary bladder	Třeboň pond area, Czech Republic	MLStef1_1F: 5′-CGTAATTCTCGGCGTATATTTGATA-3′<br>MLStef1_1R: 5′-TCAGATTTCGTTCACATTCATCCAT-3′	584/548	MT127419
				MLStef1_2F: 5′-GGCGTGTATTTGCTGATGAAATA-3′<br>MLStef1_2R: 5′-TATTAATCTATTTGGCTTGCCCTGG-3′	384/352	MT127420
				MLStef2_4F: 5′-GCGGCGTTAACAGATTATTAAGAAT-3′<br>MLStef2_4R: 5′-AGTTTTCTGCATAGTTTTAACAGCT-3′	432/401	MT127421
				MLStef3_1F: 5′-TTAAACGACAACTACCTATCACGAC-3′<br>MLStef3_1R: 5′-TGTAGTTAAATCAATCAAAGAATAA-3′	552/522	MT127422
				MLStef4_1F: 5′-AGAAGACTGGAGATTAGCTAATAAA-3′<br>MLStef4_1R: 5′-CACGCACTAATATTTTCACATTTAT-3′	516/486	MT127423
Nephrocystidium pickii	*Esox lucius*	Kidney	Třeboň pond area, Czech Republic	MLlikeStef1F: 5′-TATAGTAGAGATGGTTCCATTTGCG-3′<br>MLlikeStef1R: 5′-CCTCGGCATAGATTGTAAACTTAATGT-3′	409/374	MT127424
Tetracapsuloides bryosalmonae	*Oncorhynchus mykiss*	Trunk kidney	River Test, Hampshire, England	TbryostefFout: 5′-TTCTTATATTTAATTGTAATGTTGGGAGG-3′<br>TbryostefRout: 5′-AGTGTTTCTTTCCTACTCTAACTGA-3′	355/302	MT127425
Buddenbrockia plumatellae	*Leuciscus idus*	Kidney	Dyje river, Brno, Czech Republic	BuddStefF: 5′-ATGCTGGGAGGATTCAAGGAAGTTAAA-3′<br>BuddStefR: 5′-AGTCCACTTCCATGACATGGTAGTTTTTC-3′	300/236	MT127426
Enteromyxum leei	*Sparus aurata*	Posterior intestine	Western Mediterranean, Castellón, Spain	Eleei_Stef3out_F: 5′-CTAAACAAAATTTCGTAGCGTCTCC-3′<br>Eleei_Stef3out_R: 5′-AAGTAGTGCAGTGAATTTATAGTTC-3′	NA/525	MW498387
Enteromyxum scophthalmi	*Psetta maxima*	Anterior intestine	Galicia, NW Spain	Escop_Stef1out_F: 5′-AGAAACAAAAGAAGTTCTAATCCTCT-3′<br>Escop_Stef1out_R: 5′-TTGTGTGTTACGATTACTATTTC-3′	543/408	MW498388

				Escop_Stef2out_F: 5′-AGTTTAAGATATGCTGAAGATCGCT-3′<br>Escop_Stef2out_R: 5′-TCATAAACAAAGATAAAGAGGACTC-3′	486/427	MW498389
				Escop_Stef3out_F: 5′-ACTCAAGTATAGTAAGAACAAGATGCT-3′<br>Escop_Stef3out_R: 5′-TGAACGTGTTAATATTCAGTAAGCA-3′	463/400	MW498390

NA: not available; sequence amplified only from the cDNA template.
