# Supplementary material for: Evolutionary Analysis of Cystatins of Early-Emerging Metazoans Reveals a Novel Subtype in Parasitic Cnidarians
Source: Biology (Basel). 2021 Feb 3;10(2):110. doi: 10.3390/biology10020110 (PMC7913475; doi:10.3390/biology10020110)
Supplement: Supplementary file 1 [file biology-10-00110-s001.zip › supplements/Table S1. NGS data.pdf]

Supplementary Table 1. Next generation sequencing data of choanoflagellates and early-diverging metazoans and number of cystatin homologues mined for each species and used for phylogenetic analyses. The life cycle stage sequenced is specified (underlined) for *Edwardsiella* spp. and *Polypodium hydriforme*.

Taxonomic group/Species	GenBank identifier/Type of database	Reference	Classical stefins	Atypical stefins	Type 2 cystatins	Multi-domain cystatins
**CHOANOFLAGELLATA**						
Codosiga hollandica	GGOV01000000/TSA	(Richter, et al. 2018)	1	0	0	?
Hartaetosiga balthica	GGOO00000000/TSA	(Richter, et al. 2018)	1	0	0	?
Hartaetosiga gracilis	GGOU01000000/TSA	(Richter, et al. 2018)	1	0	0	?
Choanoeca perplexa	GGOP01000000/TSA	(Richter, et al. 2018)	1	0	0	?
Microstomoeca roanoka	GGON01000000/TSA	(Richter, et al. 2018)	1	0	0	?
Monosiga brevicollis	ABFJ01000000/WGS	(King, et al. 2008)	1	0	0	?
Mylnosiga fluctuans[x]	GGOI00000000/TSA	(Richter, et al. 2018)	1	0	0	1
Salpingoeca dolichothecata	GGOK01000000/TSA	(Richter, et al. 2018)	1	0	0	?
Salpingoeca helianthica	GGOJ01000000/TSA	(Richter, et al. 2018)	1	0	0	1
Salpingoeca infusionum	GGOW00000000/TSA	(Richter, et al. 2018)	1	0	0	?
Salpingoeca rosetta	ACSY01000000/WGS	(Fairclough, et al. 2013)	1	0	0	?
**PLACOZOA**						
Trichoplax adhaerens	ABGP01000000/WGS<br>GFSG00000000/TSA	(Srivastava, et al. 2008)<br>(Kamm, et al. 2018)	0	0	0	0
Trichoplax sp. H2 strain Panama	NOWV00000000/WGS<br>GFSF01000000/TSA	(Kamm, et al. 2018)<br>(Kamm, et al. 2018)	0	0	0	0
Trichoplax sp. strain H4 isolate TR1	SRR8193748/TSA	(Nosenko, et al. 2013)	0	0	0	0
Trichoplax sp. strain H4 isolate TR2	SRR8193747/TSA	(Nosenko, et al. 2013)	0	0	0	0
Trichoplax sp. strain H4 isolate TR3	SRR8193749/TSA	(Nosenko, et al. 2013)	0	0	0	0
Hoilungia hongkongensis	SRR5311040, SRR5311137/<br>WGS*<br>SRR5311041/TSA**	(Eitel, et al. 2018)	0	0	0	0
**PORIFERA**						
Amphimedon queenslandica	ACUQ00000000/WGS<br>LIBEST_025611/EST	(Srivastava, et al. 2010); DOE Joint Genome Institute A. queenslandica EST project	6	0	0	0
Haliclona tubifera	GFAV00000000/TSA	(Guzman and Conaco 2016)	1	0	0	0

Halisarca caerulea	GFSI00000000/TSA	(Kenny, et al. 2018)	1	0	0	0
**CTENOPHORA**						
Mnemiopsis leidyi	GFAT00000000/TSA<br>AGCP00000000/WGS	(Moroz, et al. 2014)<br>(Ryan, et al. 2013)	1	0	1	0
Hormiphora californensis	GGLO00000000/TSA	(Francis, et al. 2015)	1	0	1	0
Pleurobranchia bachei	AVPN00000000/WGS	(Moroz, et al. 2014)	0	0	1	0
Pleurobrachia pileus	LIBEST_023403/EST<br>LIBEST_022668/EST<br>SRR3417589/EST<br>SRR6074514/TSA	(Alie, et al. 2011)<br>(Philippe, et al. 2009)<br>(Simion, et al. 2017)<br>Moroz Lab	0	0	1	0
Beroe ovata	UOYG00000000/WGS	(Babonis, et al. 2018)	2	0	1	0
Beroe sp.	ERR216194/EST	(Nosenko, et al. 2013)	1	0	1	0
**CNIDARIA**						
**Staurozoa**						
Calvadosia cruxmelitensis	HAHC00000000/TSA<br>OFHS00000000/WGS	(Kayal, et al. 2018)<br>(Ohdera, et al. 2019)	1	0	0	0
Craterolophus convolvulus	HAGZ00000000/TSA	(Kayal, et al. 2018)	1	0	0	0
Haliclystus auricula	HAHA00000000/TSA	(Kayal, et al. 2018)	1	0	0	0
Haliclystus sanjuanensis	HAHB00000000/TSA	(Kayal, et al. 2018)	1	0	0	0
**Anthozoa**						
Exaiptasia pallida	LJWW00000000/WGS<br>JV124988, JV096676,<br>JV089924/TSA	(Baumgarten, et al. 2015)<br><br>(Lehnert, et al. 2012)	1	0	1	0
Nematostella vectensis	ABAV00000000/WGS<br>HADN00000000/TSA	(Putnam, et al. 2007)<br>(Babonis, et al. 2016)	2	0	1	0
Edwardsiella lineata - assembly of all life cycle stages	WGS/TSA in EdwardsiellaBase	(Stefanik, et al. 2014)	1	0	1	0
Edwardsiella carnea - Swedish isolate, parasite stage	GGGB00000000/TSA	(Dnyansagar, et al. 2018)	1	0	1	0
Edwardsiella carnea - Swedish isolate, free-living stage	GGGD00000000/TSA	(Dnyansagar, et al. 2018)	2	0	1	0
Acropora digitifera	BACK00000000/WGS<br>GIAQ01000000/TSA	(Shinzato, et al. 2015)<br>(Gajigan, et al. 2017)	3	0	1	0

	SRR3106384/TSA	(Mohamed, et al. 2016)				
Acropora hyacinthus	GDIF00000000/TSA	(Barshis, et al. 2013)	0	0	1	0
Acropora millepora	QTZP01000000/WGS<br>GHGH00000000/TSA	(Ying, et al. 2019)<br>(Ying, et al. 2019)	0	0	1	0
Acropora tenuis	IADL00000000/TSA	(Yuyama, et al. 2018)	0	0	1	0
**Cubozoa**						
Alatina alata	PUGI00000000/WGS<br>GEUJ00000000/TSA	(Ohdera, et al. 2019)<br>(Ames, et al. 2016)	1	0	2	0
Chironex fleckeri	SRR1819888/TSA	(Brinkman, et al. 2015)	0	0	1	0
Chironex yamaguchii	GHAX00000000/TSA	(Khalturin, et al. 2019)	1	0	2	0
Copula sivickisi	GHBG00000000/TSA	(Khalturin, et al. 2019)	2	0	1	0
Morbakka virulenta	RDPX00000000/WGS<br>GHAF00000000/TSA	(Khalturin, et al. 2019)<br>(Khalturin, et al. 2019)	2	0	1	0
Tripedalia cystophora	GGWE00000000/TSA<br>GHAQ00000000/TSA	(Nielsen, et al. 2019)<br>(Khalturin, et al. 2019)	1	0	3	0
**Scyphozoa**						
Aurelia aurita - Kiel Bay, Baltic Sea isolate	REGM00000000/WGS<br>GHAG00000000/TSA	(Khalturin, et al. 2019)<br>(Khalturin, et al. 2019)	2	0	1	0
Aurelia aurita - Roscoff, Pacific isolate	REGL00000000/WGS<br>GHAI00000000/TSA	(Khalturin, et al. 2019)<br>(Khalturin, et al. 2019)	3	0	1	0
Aurelia aurita - Kujukushima, aquarium, Japan isolate	GHAS00000000/TSA	(Khalturin, et al. 2019)	1	0	1	0
Aurelia aurita - White Sea isolate	GHAK00000000/TSA	(Khalturin, et al. 2019)	1	0	1	0
Aurelia aurita - Red Sea isolate	GBRG01000000/TSA	(Brekhman, et al. 2015)	2	0	1	0
Cassiopea xamachana	OLMO00000000/WGS<br>ERR1991652/TSA	(Ohdera, et al. 2019)<br>(Ohdera, et al. 2018)	0	0	1	0
Nemopilema nomurai	PEDN00000000/WGS<br>GHAR00000000/TSA	(Kim, et al., 2019)<br>(Khalturin, et al. 2019)	2	0	4	0
Cyanea capillata	SRR1930118/TSA	(Liu, et al. 2015)	0	0	1	0
Rhopilema esculentum	GFYS00000000/TSA	(Ge, et al. 2018)	0	0	1	0
Rhopilema esculentum	GEMS01000000/TSA	(Li, et al. 2017)	1	0	2	0
**Hydrozoa**						

Clytia hemisphaerica	ERP112953/WGS<br>HAMV0100000/TSA	(Leclere, et al. 2019)<br>(Leclere, et al. 2019)	1	0	1	0
Porpita porpita	GHBA00000000/TSA	(Khalturin, et al. 2019)	0	0	1	0
Velella velella	GHAZ00000000/TSA	(Khalturin, et al. 2019)	5	0	3	0
Physalia physalis	GHBB00000000/TSA	(Khalturin, et al. 2019)	1	0	1	0
Hydra vulgaris (syn. *H. magnipapillata*)	ACZU00000000/WGS	(Chapman, et al. 2010)	2	0	6	0
	ABRM00000000/WGS	(Chapman, et al. 2010)	2	0	6	0
	HAAD00000000/TSA	(Wenger and Galliot 2013)	1	0	0	0
	GGKH00000000/TSA	(Wenger, et al. 2019)	1	0	4	0
	HAAC00000000/TSA	(Wenger and Galliot 2013)	1	0	6	0
	GEVZ01000000 /TSA	(Rodrigues, et al. 2016)	0	0	4	0
	GAOL00000000/TSA	(Juliano, et al. 2014)	2	0	4	0
Millepora alcicornis	SRX323169-SRR935384/WGS<br>GFAS00000000/TSA	(Ruiz-Ramos and Baums 2014)<br>(Ortiz-Gonzalez, et al. 2017)	2	0	0	0
Podocoryna carnea	GBEH00000000/TSA<br>GCHV00000000/TSA	(Hensel, et al. 2014)<br>(Sanders and Cartwright 2015)	2	0	2	0
Turritopsis sp. SK-2016	IAAF00000000	(Hasegawa, et al. 2016)	1	0	0	0
**Polypodiozoa**						
Polypodium hydriforme - parasite stage	SRX687102-<br>SRR1558173/WGS<br>GBGH00000000/TSA	(Chang, et al. 2015)<br><br>(Shpirer, et al. 2014)	1	0	3	0
**MYXOZOA**						
**Malacosporea**						
Tetracapsuloides bryosalmonae	LIBEST_027541/EST<br>LIBEST_028454/EST<br>LIBEST_028455/EST<br>figshare***/TSA	Holland group<br>(Kumar, et al. 2015)<br>(Kumar, et al. 2015)<br>(Faber et al., 2021)	0	1	0	0
Buddenbrockia plumatellae	LIBEST_021265/EST	(Jiménez-Guri, et al. 2007)	0	1	0	0
**Myxosporea**						
Ceratonova shasta	Illumina HiSeq 2000/WGS<br>SRR6782113/TSA	Bartholomew group (unpublished)<br>(Alama-Bermejo, et al. 2020)	0	1	0	0
Enteromyxum leei	LDNA00000000/WGS	(Shpirer, et al. 2014; Chang, et al. 2015)	0	4	0	0

	Illumina NovaSeq6000 and Oxford Nanopore (FLO-MIN106)/WGS	Palenzuela group (unpublished)				
	Illumina NovaSeq6000/TSA	Palenzuela group (unpublished)				
Enteromyxum scophthalmi	Illumina NovaSeq6000 and Oxford Nanopore (FLO-MIN106)/WGS	Palenzuela group (unpublished)	0	3	0	0
	Illumina HiSeq2000 and Illumina NovaSeq6000/ TSA	Palenzuela group (unpublished)				
Kudoa iwatai	JRUX00000000/WGS<br>JRUY00000000/WGS<br>GBGI00000000/TSA	(Chang, et al. 2015)<br>(Chang, et al. 2015)<br>(Chang, et al. 2015)	0	3	0	0
Myxidium lieberkuehni	SRX9268956/TSA	(Kyslík, et al, In Press)	0	5	0	0
Nephrocystidium pickii	SRX9268957/TSA	(Kyslík, et al. In Press)	0	2	0	0
Myxobolus cerebralis	SRR628302/WGS<br>GBKL00000000/TSA	(Nesnidal, et al. 2013)<br>(Chang, et al. 2015)	0	2	0	0
Myxobolus pendula	SRX1269149 - SRR2472984, SRR2472987, SRR2472989/TSA	(Foox, et al. 2015)	0	2	0	0
Sphaeromyxa zaharoni	LDMZ00000000/WGS	(Shpirer, et al. 2014; Chang, et al. 2015)	0	3	0	0
Sphaerospora molnari	Illumina NovaSeq 6000 and Oxford Nanopore PromethION/WGS	Holzer group (unpublished)	0	2	0	0
	PRJNA522909/TSA	(Hartigan, et al. 2020)				
Thelohanellus kitauei	JWZT01000000/WGS<br>SRR1103279/TSA	(Yang, et al. 2014)<br>(Yang, et al. 2014)	0	7	0	0

[x] previously identified as *Monosiga ovata* (Carr et al. 2017); * assembly at https://bitbucket.org/molpalmuc/hoilungia-genome; ** assembly at https://bitbucket.org/molpalmuc/hoilungia-genome; *** the intersect transcriptome available from www.figshare.com, 10.6084/m9.figshare.11889672.

Abbreviations: TSA = Transcriptome Shotgun Assembly; WGS = Whole Genome Shotgun; EST = Expressed Sequence Tag.

References

Alama-Bermejo G, Meyer E, Atkinson SD, Holzer AS, Wiśniewska MM, Kolísko M, Bartholomew JL. 2020. Transcriptome-wide comparisons and virulence gene polymorphisms of host-associated genotypes of the cnidarian parasite *Ceratonova shasta* in salmonids. *Genome Biology and Evolution* 12:1258–1276.

Alie A, Leclere L, Jager M, Dayraud C, Chang PR, Le Guyader H, Queinnec E, Manuel M. 2011. Somatic stem cells express Piwi and Vasa genes in an adult ctenophore: Ancient association of "germline genes" with stemness. *Developmental Biology* 350:183-197.

Ames C, Ryan J, Bely A, Cartwright P, Collins A. 2016. A new transcriptome and transcriptome profiling of adult and larval tissue in the box jellyfish *Alatina alata*: an emerging model for studying venom, vision and sex. *BMC Genomics* 17:650.

Babonis LS, DeBiasse MB, Francis WR, Christianson LM, Moss AG, Haddock SHD, Martindale MQ, Ryan JF. 2018. Integrating embryonic development and evolutionary history to characterize tentacle-specific cell types in a ctenophore. *Molecular Biology and Evolution* 35:2940-2956.

Babonis LS, Martindale MQ, Ryan JF. 2016. Do novel genes drive morphological novelty? An investigation of the nematosomes in the sea anemone *Nematostella vectensis*. *BMC Evolutionary Biology* 16:114.

Barshis D, Ladner J, Oliver T, Seneca F, Traylor-Knowles N, Palumbi S. 2013. Genomic basis for coral resilience to climate change. *Proceedings of the National Academy of Sciences of the United States of America* 110:1387-1392.

Baumgarten S, Simakov O, Esherick LY, Liew YJ, Lehnert EM, Michell CT, Li Y, Hambleton EA, Guse A, Oates ME, et al. 2015. The genome of *Aiptasia*, a sea anemone model for coral symbiosis. *Proceedings of the National Academy of Sciences of the United States of America* 112:11893-11898.

Brekhman V, Malik A, Haas B, Sher N, Lotan T. 2015. Transcriptome profiling of the dynamic life cycle of the scypohozoan jellyfish *Aurelia aurita*. *BMC Genomics* 16:74.

Brinkman D, Jia X, Potriquet J, Kumar D, Dash D, Kvaskoff D, Mulvenna J. 2015. Transcriptome and venom proteome of the box jellyfish *Chironex fleckeri*. *BMC Genomics* 16:407.

Dnyansagar R, Zimmermann B, Moran Y, Praher D, Sundberg P, Moller L, Technau U. 2018. Dispersal and speciation: The cross Atlantic relationship of two parasitic cnidarians. *Molecular Phylogenetics and Evolution* 126:346-355.

Eitel M, Francis W, Varoqueaux F, Daraspe J, Osigus H, Krebs S, Vargas S, Blum H, Williams G, Schierwater B, et al. 2018. Comparative genomics and the nature of placozoan species. *PLoS Biology* 16: e3000032.

Faber M, Yoon S, Shaw S, de Paiva Alves E, Wang B, Qi Z, Okamura B, Hartikainen H, Secombes CJ, Holland JW. 2021. Comparative transcriptomics and host-specific parasite gene expression profiles inform on drivers of proliferative kidney disease. *Scientific Reports* 11:2149.

Fairclough S, Chen Z, Kramer E, Zeng Q, Young S, Robertson H, Begovic E, Richter D, Russ C, Westbrook M, et al. 2013. Premetazoan genome evolution and the regulation of cell differentiation in the choanoflagellate *Salpingoeca rosetta*. *Genome Biology* 14:R15.

Foox J, Ringuette M, Desser SS, Siddall ME. 2015. In silico hybridization enables transcriptomic illumination of the nature and evolution of Myxozoa. *BMC Genomics* 16:840.

Francis W, Shaner N, Christianson L, Powers M, Haddock S. 2015. Occurrence of isopenicillin-N-synthase homologs in bioluminescent ctenophores and implications for coelenterazine biosynthesis. *PLoS ONE* 10: e0128742.

Gajigan AP, Diaz LA, Conaco C. 2017. Resilience of the prokaryotic microbial community of *Acropora digitifera* to elevated temperature. *Microbiologyopen* 6: e00478.

Ge J, Liu C, Tan J, Bian L, Chen S. 2018. Transcriptome analysis of scyphozoan jellyfish *Rhopilema esculentum* from polyp to medusa identifies potential genes regulating strobilation. *Development Genes and Evolution* 228:243-254.

Guzman C, Conaco C. 2016. Comparative transcriptome analysis reveals insights into the streamlined genomes of haplosclerid demosponges. *Scientific Reports* 6: 18774.

Hasegawa Y, Watanabe T, Takazawa M, Ohara O, Kubota S. 2016. De novo assembly of the transcriptome of *Turritopsis*, a jellyfish that repeatedly rejuvenates. *Zoological Science* 33:366-371.

Hartigan A, Kosakyan A, Pecková H, Eszterbauer E, Holzer AS. 2020. Transcriptome of *Sphaerospora molnari* (Cnidaria, Myxosporea) blood stages provides proteolytic arsenal as potential therapeutic targets against sphaerosporosis in common carp. *BMC Genomics* 21: 404.

Hensel K, Lotan, Tamar, Sanders, M. S, Cartwright, Paulyn, Frank, Uri. 2014. Lineage-specific evolution of cnidarian Wnt ligands. *Evolution & Development* 16:259-269.

Chang ES, Neuhof M, Rubinstein ND, Diamant A, Philippe H, Huchon D, Cartwright P. 2015. Genomic insights into the evolutionary origin of Myxozoa within Cnidaria. *Proceedings of the National Academy of Sciences of the United States of America* 112:14912-14917.

Chapman JA, Kirkness EF, Simakov O, Hampson SE, Mitros T, Weinmaier T, Rattei T, Balasubramanian PG, Borman J, Busam D, et al. 2010. The dynamic genome of *Hydra*. *Nature* 464:592-596.

Jiménez-Guri E, Philippe H, Okamura B, Holland PWH. 2007. *Buddenbrockia* is a cnidarian worm. *Science* 317:116-118.

Juliano C, Reich A, Liu N, Gotzfried J, Zhong M, Uman S, Reenan R, Wessel G, Steele R, Lin H. 2014. PIWI proteins and PIWI-interacting RNAs function in *Hydra* somatic stem cells. *Proceedings of the National Academy of Sciences of the United States of America* 111:337-342.

Kamm K, Osigus H, Stadler P, DeSalle R, Schierwater B. 2018. *Trichoplax* genomes reveal profound admixture and suggest stable wild populations without bisexual reproduction. *Scientific Reports* 8: 11168.

Kayal E, Bentlage B, Pankey MS, Ohdera AH, Medina M, Plachetzki DC, Collins AGR, Ryan FJ. 2018. Phylogenomics provides a robust topology of the major cnidarian lineages and insights on the origins of key organismal traits. *BMC Evolutionary Biology* 18:68.

Kenny N, de Goeij J, de Bakker D, Whalen C, Berezikov E, Riesgo A. 2018. Towards the identification of ancestrally shared regenerative mechanisms across the Metazoa: A Transcriptomic case study in the Demosponge *Halisarca caerulea*. *Marine Genomics* 37:135-147.

Khalturin K, Shinzato C, Khalturina M, Hamada M, Fujie M, Koyanagi R, Kanda M, Goto H, Anton-Erxleben F, Toyokawa M, et al. 2019. Medusozoan genomes inform the evolution of the jellyfish body plan. *Nature Ecology & Evolution* 3:811-822.

Kim H-M, Weber JA, Lee N, Park SG, Cho YS, Bhak Y, Lee N, Jeon Y, Jeon S, Luria V, et al. 2019. The genome of the giant Nomura's jellyfish sheds light on the early evolution of active predation. *BMC Biology* 17:28.

King N, Westbrook M, Young S, Kuo A, Abedin M, Chapman J, Fairclough S, Hellsten U, Isogai Y, Letunic I, et al. 2008. The genome of the choanoflagellate *Monosiga brevicollis* and the origin of metazoans. *Nature* 451:783-788.

Kumar G, Abd-Elfattah A, El-Matbouli M. 2015. Identification of differentially expressed genes of brown trout (*Salmo trutta*) and rainbow trout (*Oncorhynchus mykiss*) in response to *Tetracapsuloides bryosalmonae* (Myxozoa). *Parasitology Research* 114:929-939.

Kyslík J, Kosakyan A, Nenarokov S, Holzer AS, Fiala I. 2021. Myxozoan minicollagen gene repertoire is not simplified by the parasitic lifestyle: Computational identification of a novel myxozoan minicollagen gene. *BMC Genomics*. In Press.

Leclere L, Horin C, Chevalier S, Lapebie P, Dru P, Peron S, Jager M, Condamine T, Pottin K, Romano S, et al. 2019. The genome of the jellyfish *Clytia hemisphaerica* and the evolution of the cnidarian life-cycle. *Nature Ecology & Evolution* 3:801-810.

Lehnert EM, Burriesci MS, Pringle JR. 2012. Developing the anemone *Aiptasia* as a tractable model for cnidarian-dinoflagellate symbiosis: the transcriptome of aposymbiotic *A. pallida*. *BMC Genomics* 13:271.

Li YF, Zhou ZC, Tian ML, Tian Y, Dong Y, Li SL, Liu WD, He CB. 2017. Exploring single nucleotide polymorphism (SNP), microsatellite (SSR) and differentially expressed genes in the jellyfish (*Rhopilema esculentum*) by transcriptome sequencing. *Marine Genomics* 34:31-37.

Liu G, Zhou Y, Liu D, Wang Q, Ruan Z, He Q, Zhang L. 2015. Global transcriptome analysis of the tentacle of the jellyfish *Cyanea capillata* using deep sequencing and expressed sequence tags: Insight into the toxin- and degenerative disease-related transcripts. *PLoS ONE* 10: e0142680.

Mohamed AR, Cumbo V, Harii S, Shinzato C, Chan CX, Ragan MA, Bourne DG, Willis BL, Ball EE, Satoh N, et al. 2016. The transcriptomic response of the coral *Acropora digitifera* to a competent *Symbiodinium* strain: the symbiosome as an arrested early phagosome. *Molecular Ecology* 25:3127-3141.

Moroz L, Kocot K, Citarella M, Dosung S, Norekian T, Povolotskaya I, Grigorenko A, Dailey C, Berezikov E, Buckley K, et al. 2014. The ctenophore genome and the evolutionary origins of neural systems. *Nature* 510:109-114.

Nesnidal M, Helmkampf M, Bruchhaus I, El-Matbouli M, Hausdorf B. 2013. Agent of whirling disease meets orphan worm: Phylogenomic analyses firmly place Myxozoa in Cnidaria. *PLoS ONE* 8: e54576.

Nielsen S, Koch T, Hauser F, Garm A, Grimmelikhuijzen C. 2019. De novo transcriptome assembly of the cubomedusa *Tripedalia cystophora*, including the analysis of a set of genes involved in peptidergic neurotransmission. *BMC Genomics* 20:175.

Nosenko T, Schreiber F, Adamska M, Adamski M, Eitel M, Hammel J, Maldonado M, Muller WEG, Nickel M, Schierwater B, et al. 2013. Deep metazoan phylogeny: When different genes tell different stories. *Molecular Phylogenetics and Evolution* 67:223-233.

Ohdera A, Ames CL, Dikow RB, Kayal E, Chiodin M, Busby B, La S, Pirro S, Collins AG, Medina M, et al. 2019. Box, stalked, and upside-down? Draft genomes from diverse jellyfish (Cnidaria, Acraspeda) lineages: *Alatina alata* (Cubozoa), *Calvadosia cruxmelitensis* (Staurozoa), and *Cassiopea xamachana* (Scyphozoa). *GigaScience* 8:7.

Ohdera AH, Abrams MJ, Ames CL, Baker DM, Suescun-Bolivar LP, Collins AG, Freeman CJ, Gamero-Mora E, Goulet TL, Hofmann DK, et al. 2018. Upside-down but headed in the right direction: Review of the highly versatile *Cassiopea xamachana* system. *Frontiers in Ecology and Evolution* 6.

Ortiz-Gonzalez I, Rivera-Vicens R, Schizas N. 2017. De novo transcriptome assembly of the hydrocoral *Millepora alcicornis* (branching fire coral) from the Caribbean. *Marine Genomics* 32:27-30.

Philippe H, Derelle R, Lopez P, Pick K, Borchiellini C, Boury-Esnault N, Vacelet J, Renard E, Houliston E, Queinnec E, et al. 2009. Phylogenomics revives traditional views on deep animal relationships. *Current Biology* 19:706-712.

Putnam NH, Srivastava M, Hellsten U, Dirks B, Chapman J, Salamov A, Terry A, Shapiro H, Lindquist E, Kapitonov VV, et al. 2007. Sea anemone genome reveals ancestral eumetazoan gene repertoire and genomic organization. *Science* 317:86-94.

Richter D, Fozouni P, Eisen M, King N. 2018. Gene family innovation, conservation and loss on the animal stem lineage. *Elife* 7: e34226.

Rodrigues M, Ostermann T, Kremeser L, Lindner H, Beisel C, Berezikov E, Hobmayer B, Ladurner P. 2016. Profiling of adhesive-related genes in the freshwater cnidarian *Hydra magnipapillata* by transcriptomics and proteomics. *Biofouling* 32:1115-1129.

Ruiz-Ramos D, Baums I. 2014. Microsatellite abundance across the Anthozoa and Hydrozoa in the phylum Cnidaria. *BMC Genomics* 15: 939.

Ryan J, Pang K, Schnitzler C, Nguyen A, Moreland R, Simmons D, Koch B, Francis W, Havlak P, Smith S, et al. 2013. The genome of the ctenophore *Mnemiopsis leidyi* and its implications for cell type evolution. *Science* 342: 1242592.

Sanders S, Cartwright P. 2015. Interspecific differential expression analysis of RNA-Seq data yields insight into life cycle variation in hydractiniid hydrozoans. *Genome Biology and Evolution* 7:2417-2431.

Shinzato C, Mungpakdee S, Arakaki N, Satoh N. 2015. Genome-wide SNP analysis explains coral diversity and recovery in the Ryukyu Archipelago. *Scientific Reports* 5: 18211.

Shpirer E, Chang E, Diamant A, Rubinstein N, Cartwright P, Huchon D. 2014. Diversity and evolution of myxozoan minicollagens and nematogalectins. *BMC Evolutionary Biology* 14:205.

Simion P, Philippe H, Baurain D, Jager M, Richter DJ, Di Franco A, Roure B, Satoh N, Queinnec E, Ereskovsky A, et al. 2017. A Large and consistent phylogenomic dataset supports sponges as the sister group to all other animals. *Current Biology* 27:958-967.

Srivastava M, Begovic E, Chapman J, Putnam N, Hellsten U, Kawashima T, Kuo A, Mitros T, Salamov A, Carpenter M, et al. 2008. The *Trichoplax* genome and the nature of placozoans. *Nature* 454:955-U919.

Srivastava M, Simakov O, Chapman J, Fahey B, Gauthier M, Mitros T, Richards G, Conaco C, Dacre M, Hellsten U, et al. 2010. The *Amphimedon queenslandica* genome and the evolution of animal complexity. *Nature* 466:720-U723.

Stefanik DJ, Lubinski TJ, Granger BR, Byrd AL, Reitzel AM, DeFilippo L, Lorenc A, Finnerty JR. 2014. Production of a reference transcriptome and transcriptomic database (EdwardsiellaBase) for the lined sea anemone, *Edwardsiella lineata*, a parasitic cnidarian. *BMC Genomics* 15:19.

Wenger Y, Buzgariu W, Perruchoud C, Loichot G, Galliot B. 2019. Generic and context-dependent gene modulations during *Hydra* whole body regeneration. bioRxiv:587147.

Wenger Y, Galliot B. 2013. RNAseq versus genome-predicted transcriptomes: a large population of novel transcripts identified in an Illumina-454 *Hydra* transcriptome. *BMC Genomics* 14:204.

Yang YL, Xiong J, Zhou ZG, Huo FM, Miao W, Ran C, Liu YC, Zhang JY, Feng JM, Wang M, et al. 2014. The genome of the myxosporean *Thelohanellus kitauei* shows adaptations to nutrient acquisition within its fish host. *Genome Biology and Evolution* 6:3182-3198.

Ying H, Hayward DC, Cooke I, Wang W, Moya A, Siemering KR, Sprungala S, Ball EE, Forêt S, Miller DJ. 2019. The whole-genome sequence of the coral *Acropora millepora*. *Genome Biology Evolution* 11:1374-1379.

Yuyama I, Ishikawa M, Nozawa M, Yoshida M, Ikeo K. 2018. Transcriptomic changes with increasing algal symbiont reveal the detailed process underlying establishment of coral-algal symbiosis. *Scientific Reports* 8: 16802.
