# Supplementary material for: Evolutionary Analysis of Cystatins of Early-Emerging Metazoans Reveals a Novel Subtype in Parasitic Cnidarians
Source: Biology (Basel). 2021 Feb 3;10(2):110. doi: 10.3390/biology10020110 (PMC7913475; doi:10.3390/biology10020110)
Supplement: Supplementary file 1 [file biology-10-00110-s001.zip › supplements/Fig S1 ML and BI tree with nodal supports.pdf]

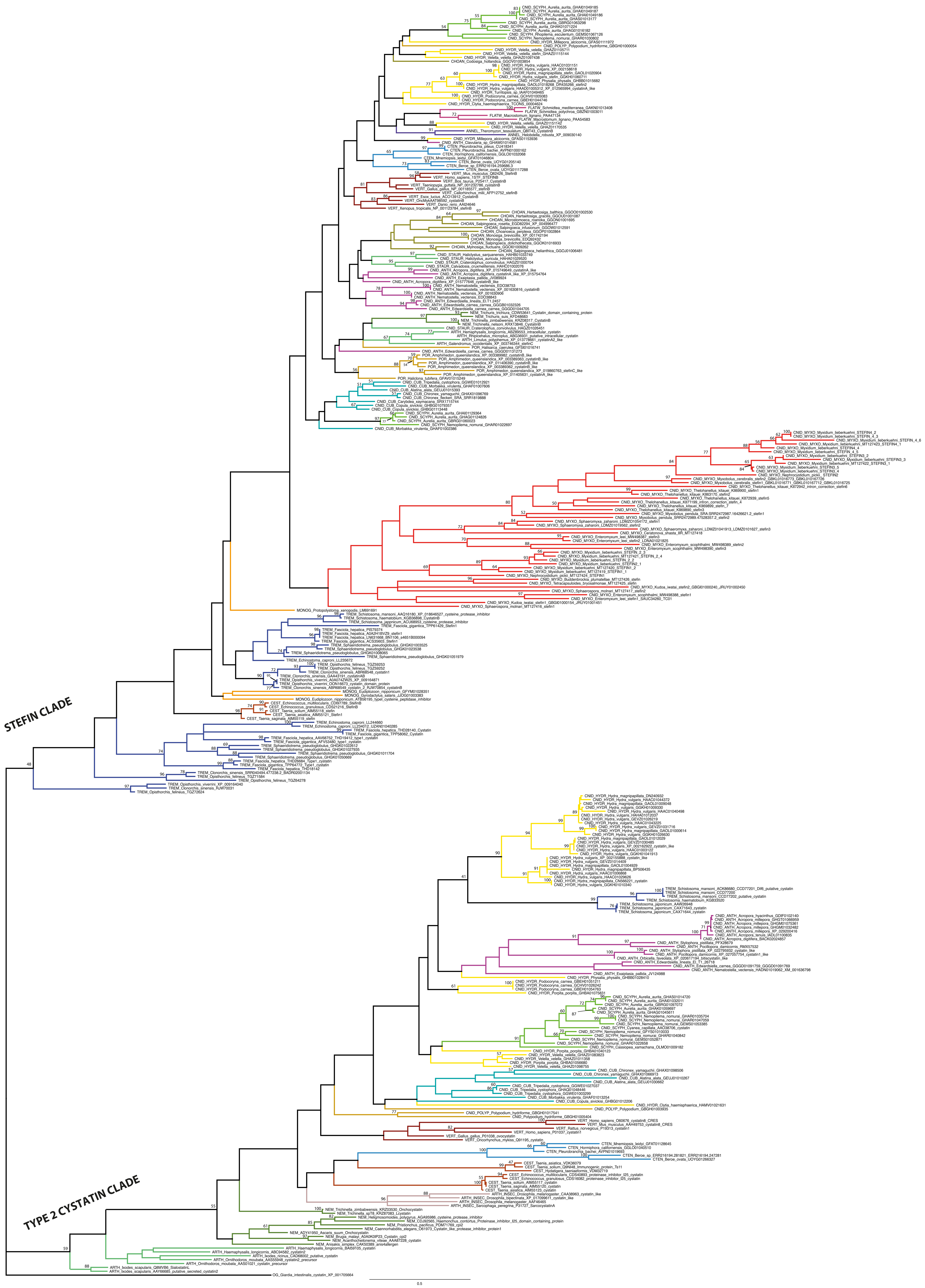

Maximum likelihood phylogenetic tree of the cystatin superfamily represented in the Metazoa and Choanoflagellata with nodal supports calculated from 1000 bootstrap replicates shown at nodes with >50% support. *Giardia* cystatin was used as the outgroup. Colours of branches correspond to Figure 3.

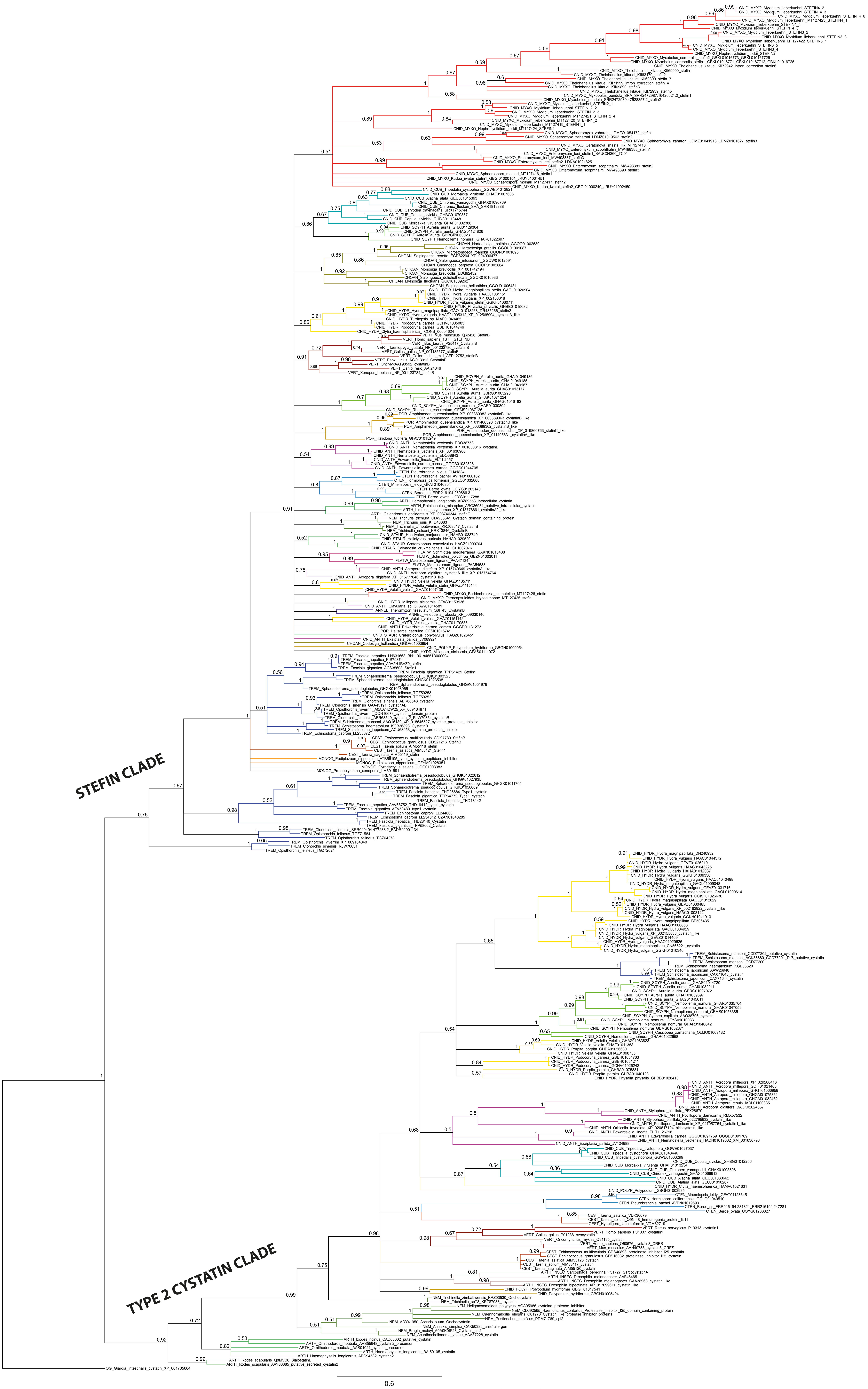

Bayesian inference phylogenetic tree of the cystatin superfamily represented in the Metazoa and Choanoflagellata with posterior probabilbilites shown at each node. *Giardia* cystatin was used as the outgroup. Colours of branches correspond to Figure 3.
