# Supplementary material for: Evolutionary Analysis of Cystatins of Early-Emerging Metazoans Reveals a Novel Subtype in Parasitic Cnidarians
Source: Biology (Basel). 2021 Feb 3;10(2):110. doi: 10.3390/biology10020110 (PMC7913475; doi:10.3390/biology10020110)
Supplement: Supplementary file 1 [file biology-10-00110-s001.zip › supplements/Methods S1. PCR verification.pdf]

Verification of selected myxozoan stefin sequences

Origin and type of samples of each myxozoan species used for PCR verification is specified in Table S2. Samples were processed freshly or fixed in 90% ethanol (DNA) and in RNAprotect Cell Reagent (Qiagen, Germany) or RNA-later (Ambion, USA) (RNA) for subsequent DNA and RNA extraction. Tissue samples were homogenized in TRIreagent (Sigma, Germany) or in Lysing Matrix D tubes (MP Biomedicals, USA) containing 1 ml of TRIreagent using a FastPrep-24 homogenizer (MP Biomedicals, USA; 2 cycles of 30 s at 6 m/s). Total DNA was extracted from fresh or fixed samples using the standard phenol-chloroform protocol after overnight digestion with proteinase K (Serva, Germany) at 55℃ or using the High Pure PCR Template Preparation Kit (Roche Diagnostic GmbH, Germany) following manufacturer's instructions. Total RNA was extracted using the phenol-chloroform protocol or with the Nucleospin RNA Kit (Macherey-Nagel, Germany) or MagMax-96 for Microarray Total RNA Isolation kit (Ambion, USA) following manufacturer's instructions. Purified genomic DNA or RNA was quantified using a Nanodrop spectrophotometer (NanoDrop Technologies, USA). Prior to reverse transcription, 500 ng of RNA were treated with DNaseI amplification grade (Invitrogen, USA) and reverse transcribed using the High-Capacity cDNA Archive Kit (Applied Biosystems, USA), the Transcriptor High Fidelity cDNA Synthesis Kit (Roche, Switzerland) or using Bioscript (Bioline, UK) following manufacturer's instructions.

PCR amplifications of *Sphaerospora molnari, Ceratonova shasta, Myxidium lieberkuehni, Nephrocystidium pickii*, and *Buddenbrockia plumatellae* stefins were performed using the AmpOne HS-Taq premix (GeneAll Biotechnology, South Korea). Each PCR contained 10 µl premix, 0.5 µl each primer (25 pmol), 8 µl Milli-Q H_2O and 10 to 150 ng DNA/cDNA. Primer combinations for each gene and species are summarized in Supplementary Table 2. Cycling parameters: 95℃ for 3 min, the 30 cycles of 95℃ for 1 min, 55° for 1 min, 72℃ for 1 min), followed by 72℃ for 7 min. PCR products were purified using the Gel/PCR DNA Fragments Extraction Kit (Geneaid Biotech Ltd, Taiwan). Amplified fragments were cloned into the pDrive Vector with the PCR Cloning Kit (Qiagen, Germany) and transformed into DH5α chemically competent *Escherichia coli* cells (Thermo Fisher Scientific, USA). Plasmid DNA was extracted using the High Pure Plasmid Isolation Kit (Roche, Germany) and three clones were commercially sequenced using the M13 forward and reverse vector primers (SeqMe, Czech Republic).

PCR amplification of *Enteromyxum leei and E. scophthalmi* stefins was performed using DNA and/or cDNA templates and the Platinum™ Taq DNA Polymerase High Fidelity using primer combinations listed in Supplementary Table 2. Cycling parameters: 94℃ for 3 min, the 35 cycles of 94℃ for 45 sec, 55° for 30 sec, 68℃ for 1 min), followed by 72℃ for 10 min. Ligation and cloning was done with TOPO TA cloning kit (Thermo Fisher Scientific, USA) and competent *E. coli* cells, following the manufacturer’s instructions. Several colonies (up to 6) were tested by PCR to ensure that the colony had incorporated the plasmid. Plasmid DNA was extracted using PureLink™ Quick Plasmid Miniprep Kit (Invitrogen, USA) and up to 6 colonies were sequenced at the in-house sequencing platform of the Institute for Plant Molecular and Cellular Biology (Spain) using the M13 forward and reverse vector primers.

PCR amplification of *Tetracapsuloides bryosalmonae* stefin was done from cDNA template using BIOTAQ™ DNA polymerase, dNTPs (10mM), $MgCl_2$ (80mM) and primer combinations listed in Supplementary Table 2. Cycling parameters: 95℃ for 3 min, the 25 cycles of 95℃ for 30 sec, 58˚ for 30 sec, 72℃ for 1 min), followed by 72℃ for 4 min. The PCR products were purified using the PureLink™ PCR Purification Kit (Thermo Fisher Scientific, USA) and cloned into pGEM-T easy vector (Promega, USA), transfected into α-Select Silver Competent Cells (Bioline, UK) and screened using the M13-Uni/Rev vector primers. The positive clones were purified and commercially sequenced using the M13 forward and reverse vector primers by Eurofins Genomics (Germany).
