# Supplementary material for: Evolutionary Analysis of Cystatins of Early-Emerging Metazoans Reveals a Novel Subtype in Parasitic Cnidarians
Source: Biology (Basel). 2021 Feb 3;10(2):110. doi: 10.3390/biology10020110 (PMC7913475; doi:10.3390/biology10020110)
Supplement: Supplementary file 1 [file biology-10-00110-s001.zip › supplements/Table S3. Taxa used for phylog analyses.pdf]

Table S3. The list of groups/species used for phylogenetic analyses. Parasite species are shaded red, free-living species are shaded green and species with both parasite and free-living life cycle stages are shaded yellow.

EARLY-DIVERGING METAZOAN GROUPS	DERIVED METAZOAN GROUPS
**PORIFERA**	**TREMATODA**
Amphimedon queenslandica	*Clonorchis sinensis*
Haliclona tubifera	*Echinostoma caproni*
Halisarca caerulea	*Fasciola gigantica*
**CTENOPHORA**	*Fasciola hepatica*
Beroe ovata	*Opisthorchis felineus*
Beroe sp.	*Opisthorchis viverrini*
Hormiphora californensis	*Schistosoma haematobium*
Mnemiopsis leidyi	*Schistosoma japonicum*
Pleurobrachia pileus	*Schistosoma mansoni*
Pleurobranchia bachei	*Sphaeridiotrema pseudoglobulus*
**CNIDARIA/STAUROZOA**	**NEMATODA**
Calvadosia cruxmelitensis	*Acanthocheilonema viteae*
Craterolophus convolvulus	*Anisakis simplex*
Haliclystus auricula	*Ascaris suum*
Haliclystus sanjuanensis	*Brugia malayi*
**CNIDARIA/ANTHOZOA**	*Caenorhabditis elegans*
Acropora digitifera	*Haemonchus contortus*
Acropora hyacinthus	*Heligmosomoides polygyrus*
Acropora millepora	*Pristionchus pacificus*
Acropora tenuis	*Trichinella* sp.
Clavularia sp.	*Trichinella nelsoni*
Edwardsiella carnea	*Trichinella zimbabwensis*
Edwardsiella lineata	*Trichuris suis*
Exaiptasia pallida	*Trichuris trichiura*
Nematostella vectensis	**MONOGENEA**
Orbicella faveolata	*Eudiplozoon nipponicum*
Pocillopora damicornis	*Gyrodactylus salaris*
Stylophora pistillata	*Protopolystoma xenopodis*
**CNIDARIA/SCYPHOZOA**	**CESTODA**
Aurelia aurita	*Echinococcus granulosus*
Cassiopea xamachana	*Echinococcus multilocularis*
Cyanea capillata	*Hydatigera taeniaeformis*
Nemopilema nomurai	*Taenia asiatica*
Rhopilema esculentum	*Taenia saginata*
**CNIDARIA/CUBOZOA**	*Taenia solium*
Alatina alata	**ANNELIDA**
Copula sivickisi	*Helobdella robusta*
Chironex fleckeri	*Theromyzon tessulatum*
Chironex yamaguchii	**TURBELLARIA**
Morbakka virulenta	*Macrostomum lignano*
Tripedalia cystophora	*Schmidtea mediterranea*
**CNIDARIA/HYDROZOA**	*Schmidtea polychroa*
Clytia hemisphaerica	**ARTHROPODA/CHELICERATA**
Hydra vulgaris (syn. *H. magnipapillata*)	*Galendromus occidentalis*
Millepora alcicornis	*Haemaphysalis longicornis*
Physalia physalis	*Ixodes ricinus*
Podocoryna carnea	*Ixodes scapularis*
Porpita porpita	*Limulus polyphemus*
Turritopsis sp.	*Ornithodoros moubata*

Velella velella	*Rhipicephalus microplus*
**CNIDARIA/POLYPODIOZOA**	**ARTHROPODA/INSECTA**
Polypodium hydriforme	*Drosophila bipectinata*
**CNIDARIA/MYXOZOA**	*Drosophila melanogaster*
Buddenbrockia plumatellae	*Sarcophaga peregrina*
Ceratonova shasta	**VERTEBRATA**
Enteromyxum leei	*Bos taurus*
Enteromyxum scophthalmi	*Callorhinchus milii*
Kudoa iwatai	*Danio rerio*
Myxidium lieberkuehni	*Esox lucius*
Myxobolus cerebralis	*Gallus gallus*
Myxobolus pendula	*Homo sapiens*
Nephrocystidium pickii	*Mus musculus*
Sphaeromyxa zaharoni	*Oncorchynchus mykiss*
Sphaerospora molnari	*Rattus norvegicus*
Tetracapsuloides bryosalmonae	*Taeniopygia guttata*
Thelohanellus kitauei	*Xenopus tropicalis*
**NON-METAZOAN GROUPS**	**OUTGROUP**
**CHOANOFLAGELLATA**	**EXCAVATA**
Codosiga hollandica	*Giardia intestinalis*
Hartaetosiga balthica	
Hartaetosiga gracilis	
Choanoeca perplexa	
Microstomoeca roanoka	
Monosiga brevicollis	
Mylnosiga fluctuans	
Salpingoeca dolichothecata	
Salpingoeca helianthica	
Salpingoeca infusionum	
Salpingoeca rosetta	
